# Attempts to Image the Early Inflammatory Response during Infection with the Lymphatic Filarial Nematode *Brugia pahangi* in a Mouse Model

**DOI:** 10.1371/journal.pone.0168602

**Published:** 2016-12-16

**Authors:** Elmarie Myburgh, Ryan Ritchie, Amy Goundry, Kerry O’Neill, Francesco Marchesi, Eileen Devaney

**Affiliations:** 1 Wellcome Trust Centre for Molecular Parasitology, Institute of Infection, Immunity and Inflammation, College of Medical, Veterinary and Life Sciences, University of Glasgow, Glasgow, United Kingdom; 2 Institute of Biodiversity, Animal Health and Comparative Medicine, University of Glasgow, Garscube Estate, Glasgow, United Kingdom; 3 School of Veterinary Medicine, University of Glasgow, Garscube Estate, Glasgow; University of Illinois, UNITED STATES

## Abstract

Helminth parasites remain a major constraint upon human health and well-being in many parts of the world. Treatment of these infections relies upon a very small number of therapeutics, most of which were originally developed for use in animal health. A lack of high throughput screening systems, together with limitations of available animal models, has restricted the development of novel chemotherapeutics. This is particularly so for filarial nematodes, which are long-lived parasites with a complex cycle of development. In this paper, we describe attempts to visualise the immune response elicited by filarial parasites in infected mice using a non-invasive bioluminescence imaging reagent, luminol, our aim being to determine whether such a model could be developed to discriminate between live and dead worms for *in vivo* compound screening. We show that while imaging can detect the immune response elicited by early stages of infection with L3, it was unable to detect the presence of adult worms or, indeed, later stages of infection with L3, despite the presence of worms within the lymphatic system of infected animals. In the future, more specific reagents that detect secreted products of adult worms may be required for developing screens based upon live imaging of infected animals.

## Introduction

Drug discovery against parasitic organisms has undergone something of a renaissance in recent years with the impact of the London Declaration (https://www.gov.uk/government) and the involvement of funding agencies such as the Bill and Melinda Gates Foundation and Drugs for Neglected Diseases *initiative* (DND*i*). The World Health Organisation (WHO) aim of eradicating several parasitic infections, such as the lymphatic filariae (http://www.filariasis.org), together with the increasing threat of drug resistance [1,2] underpins the requirement for novel chemotherapeutics. For efficient drug discovery a high throughput screen (HTS) of large chemical libraries is a desirable first step for the detection of chemotypes with activity against the organism in question.”Hits” then undergo chemical modification to increase their drug-like properties whilst maintaining their selective anti-parasite activity. For protozoan parasites such as *Plasmodium*, *Leishmania* or *Trypanosoma* species, various HTS have been devised that facilitate the identification of novel agents [3,4]. For helminth parasites, there are few comparable methods that allow screening of large chemical libraries (thousands of compounds) to identify those with anthelmintic activity. In part this relates to differences in the basic biology of helminth and protozoan parasites; many protozoans can be cultured *in vitro* and will replicate under such conditions providing a relatively simple readout of drug activity. In contrast, helminth parasites are notoriously difficult to culture [5] and do not replicate *in vitro*.

For parasites such as filarial worms, there is a pressing need for drugs with activity against adult worms to help achieve the goal of elimination. Most primary screens for anti-filarial compounds rely upon motility assays, which can be scaled up to test a few hundred compounds at best. Recent studies have resulted in significant improvements by the use of video recording and computational algorithms that provide an objective measure of drug efficacy [6,7]. Once compounds are identified from such *in vitro* screens, they are commonly tested in various animal models prior to further development. For filarial worms, the paucity of small rodent systems for subsequent *in vivo* testing of potential hits further impedes drug discovery.

For parasitic protozoa, the ability to transfect parasites with plasmid constructs expressing fluorescent or bioluminescent reporter proteins, such as mCherry or luciferase, has provided a useful tool for the *in vivo* visualization of parasites. Using this technology it is possible to track parasites within the live animal and to demonstrate the efficacy of various drugs or immunological interventions over time in the same cohort of animals (see [8,9], for example). Applying transgenic technology to parasitic helminths is significantly more difficult, although there have been notable successes with *Schistosoma* [10] and *Strongyloides* species [11]. However, genetic modification of filarial nematodes pose particular problems, as the developmental cycle is long (a minimum of 3 months) and complex, involving an arthropod vector, and there are no free-living stages that could be used to amplify the numbers of transfected worms [12]. In this study, we investigated an alternative approach to visualizing filarial worms in live animals by applying non-invasive bioluminescence imaging of the host response to infection. In inflammatory conditions, probes such as luminol and lucigenin have proved to be useful tools for distinguishing the differential role of immune cells in various pathologies [13]. For example, luminol is activated by the myeloperoxidase activity of neutrophils [14], while lucigenin requires NADPH activity, characteristic of macrophages [13]. A similar concept was applied to image the eosinophilic response elicited by *Schistosoma mansoni* infection in the tissues, only in this case the mice expressed an inducible luciferase reporter gene driven by an eosinophil-specific promoter [15]. Here, we demonstrate that while whole mouse *in vivo* imaging using luminol as a substrate can detect the early stages of infection with the third stage larvae (L3) of *Brugia pahangi*, the current limitations of the system are such that its usefulness in drug screening is restricted.

## Materials and Methods

### Ethics statement

All animal protocols were carried out in accordance with the guidelines of the UK Home Office, under the Animal (Scientific Procedures) Act 1986, following approval by the University of Glasgow Ethical Review Panel. Experiments were performed under the authority of the UK Home Office, project number 60/4448. Animals were anaesthetized for surgery using a mixture of Ketamine and Dormitor and for imaging using an isoflurane anaesthesia unit. At the end of the experiments animals were sacrificed by cervical dislocation or CO_2_ inhalation.

### Parasites and infection of mice

The *Brugia pahangi* life cycle was maintained by serial passage through mosquitoes (*Aedes aegypti*, Refm) and jirds, *Meriones unguiculatus*, as described previously [16]. All mouse experiments were performed with male BALB/c mice, 6–8 weeks of age, and animals were maintained in filter top cages for the duration of the experiments. A transplant model was used to study the response elicited by *B*. *pahangi* adult worms, exactly as described previously [17,18]. In brief, adult female worms were removed from the peritoneal cavity of infected jirds, rinsed in HBSS and 10 worms transplanted into the peritoneal cavity of each of four anaesthetized BALB/c mice. Four sham control animals that underwent the same procedure, but had no adult worms implanted were included for imaging. To distinguish between worm and surgery-related responses six control animals that were injected intraperitoneally with HBSS 12 days before imaging but underwent no surgery were included for analysis. Imaging (described in “IVIS reagent and imaging”) was performed on d17 and d27 post-transplantation.

For larvae infections mice were injected with L3 of *B*. *pahangi* harvested from mosquitoes infected 9 days previously. L3 were washed in sterile Hanks Balanced Salt Solution (HBSS, Invitrogen) and counted into groups of 50. For the intraperitoneal (ip) infection, L3 were taken up into approximately 250 μl of sterile HBSS in a standard syringe and injected into 12 mice while six control animals received an equivalent volume of HBSS. Six infected and six control mice were imaged on day 7, 12 and 18 p.i. (as described below) while the remaining six infected mice were sacrificed at day 6 p.i. to assess larvae and host cells in the peritoneal cavity. For intra-dermal (footpad) injection, L3 were taken up into a maximum volume of 50 μl in a single use Myjector U-100 insulin syringe (Terumo Medical Corporation, MD, USA) and injected into the right-hand side (RHS) footpad of seven mice. The left-hand side (LHS) footpad received an equivalent volume of HBSS. Syringes were flushed with HBSS following injection and the remaining L3 counted, as 100% of the worms are rarely injected. Mice were imaged on day 4 and 11 p.i. as described below. In a further experiment, groups of three mice were injected via the footpad with 5, 25 or 50 L3 and imaged on day 5 and 11 p.i.

### IVIS reagents and imaging

Luminol sodium salt (Sigma-Aldrich) was prepared in PBS at 50 mg/ml and frozen until required for imaging. At the indicated time points, luminol was injected subcutaneously (sc) into the neck at 200 mg/kg body weight. Mice were imaged 20 min later under isoflurane anaesthesia using an IVIS Spectrum imaging system (Caliper Life Sciences).

Images were acquired in bioluminescence mode with an open emission filter, for 1 min exposures, large binning, and 1 f/stop, and captured with a charge-coupled device (CCD) camera. Analysis was performed using Living Image software (Caliper Life Sciences). The absolute unit of photon emission was given as radiance (photons/second/cm^2^/steradian). Regions of interest (ROIs) were manually selected over the abdomen or foot, depending on the site of infection, to quantify photon emission as total flux in photons per second (photons/sec).

### Histological evaluation

On day 7, 14 and 21 post-injection of L3 into the footpad, mice (four per time point) were euthanised and the limbs processed for histological examination following fixation in 10% neutral buffered formal saline. Tissues were decalcified in 4% EDTA/formalin and then processed to paraffin blocks. Sections approximately 2.5 μm thick were cut and stained with haematoxylin and eosin following standard procedures. The involvement of eosinophils in the inflammatory response in infected limbs was investigated by staining sections using modified haematoxylin and eosin and Congo Red staining protocols, as described [19].

### Cytokine measurements by bead array

At days 7 and 14 post-infection (p.i.), mice (five infected and five naive per time point) were euthanised and the popliteal lymph nodes (popLN) were removed and processed for *in vitro* culture essentially as described previously [20]. In brief, LN were disrupted by passage through a 0.22 μm cell strainer and 1x10^6^ cells/ml were cultured in RPMI-1640 containing L-glutamine, sodium bicarbonate (Sigma) and 10% heat-inactivated fetal bovine serum for 48 h at 37°C in an atmosphere of 5% CO_2_ in air. Cells were cultured in the presence or absence of *Brugia* antigen at a concentration of 10 μg/ml. Antigen was prepared from adult worms exactly as described previously [20]. Supernatants were collected after 48 h of culture and processed for cytokine analysis using a Bio-Plex Pro mouse cytokine assay kit (Bio-Rad).

### Statistical analysis

All *p* values were determined with Prism software (GraphPad Software Inc) using the test described in the figure legends.

## Results

### IVIS following intra-peritoneal infections

**Fig 1:**
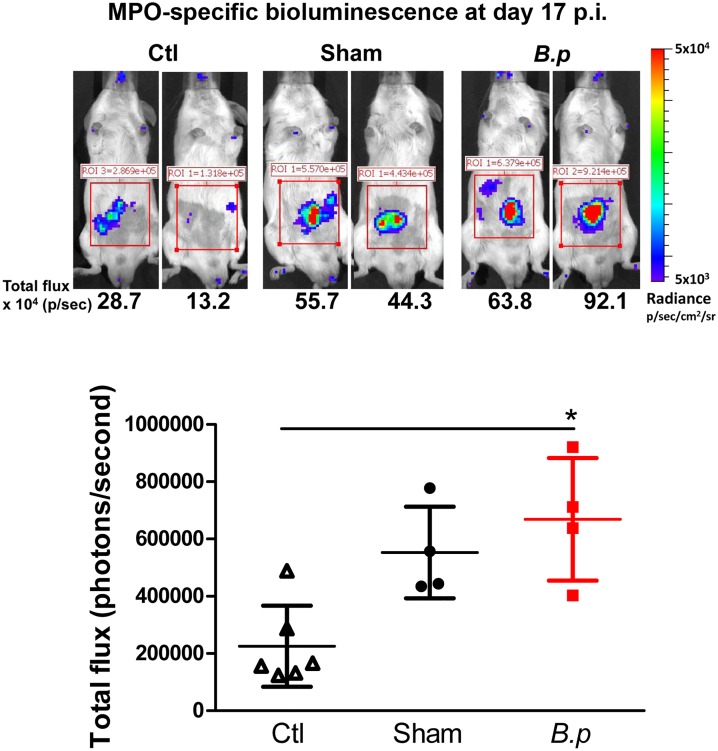
*In vivo* imaging of MPO-specific bioluminescence in mice implanted with adult *B*. *pahangi*. BALB/c mice were infected intraperitoneally by transplantation with 10 female adult *B*. *pahangi (B*.*p)*. Sham-operated mice (Sham) received no worms and control mice (Ctl) received an intraperitoneal injection of HBSS but underwent no surgery. On day 17 post-infection mice were imaged using an IVIS spectrum 20 minutes after subcutaneous injection of 200 mg/kg luminol. Representative images of 2 mice per group are shown. The colour scale indicates bioluminescence radiance in photons/second/cm^2^/steradian. Graphs show the bioluminescence total flux (in photons/second) within the same abdominal region of interest. Each symbol shows the total flux for a single mouse, lines indicate the means (n = 4–6 mice) and error bars show SD (*p < 0.05 using a one-way ANOVA with Dunn’s post-test).

As the greatest need for novel anti-filarial compounds is for drugs with macrofilaricidal activity, we initially assessed the utility of an *in vivo* imaging system (IVIS) for detecting live adult worms following transplantation. In this experiment, four BALB/mice were infected by ip transplantation with 10 adult female *B*. *pahangi*. Sham control animals were treated identically (undergoing a sham operation) except that they received no worms, and surgery control animals were injected ip with HBSS but underwent no surgery. Mice were allowed to recover fully from the operation and then imaged using luminol on day 17 following infection. Luminol was injected subcutaneously into the neck to minimize potential inflammatory responses in the abdominal region due to tissue damage caused by substrate injection. Although the luminescent signal obtained was restricted to the abdominal cavity, the overall signal was relatively low (mean 6.7 x 10^5^ ± 2.1 x 10^5^ photons per second in infected mice compared to 2.3 x 10^5^ ± 1.4 x 10^5^ photons per second in six HBSS-injected mice, *p*< 0.05), and there was no difference in intensity from infected and sham control animals (mean 5.6 x 10^5^ ± 1.7 x 10^5^) (Fig 1). The increase in bioluminescence in the sham-operated compared to HBSS-injected mice (although not significant) suggests that the surgery was sufficient to elicit a response detectable by IVIS. This increase in signal may mask small changes in inflammation elicited by the presence of worms. Imaging was also carried out at day 27 p.i., but no discrimination was obtained between sham-operated and infected animals.

As the problem with adult infections was most likely a result of a response to the invasive procedure required to implant the worms, we next infected 12 BALB/c mice by ip inoculation with 50 L3, while a further six animals received HBSS alone. At 7, 12 and 18 days p.i., six control and six infected animals were imaged following subcutaneous (sc) injection of luminol. A significant increase in luminol-specific signal was observed for *B*. *pahangi*-infected mice at day 7 (mean 5.012 x 10^5^ ± 3.8 x 10^5^ after luminol injection compared to 9.7 x 10^4^ ± 9.2 x 10^4^ without luminol injection). However no specificity in the luminescent signal was detected at any time point, as mice receiving an injection of HBSS alone gave similar signals (S1 Fig). To ensure that the infection was successful, the remaining six animals in this experiment were sacrificed at day 6 p.i. and the numbers of developing larvae were assessed by lavage of the peritoneal cavity with HBSS. Worm recoveries were variable 0–79% (mean 35±27%) in keeping with previous data from this model system [18], but five out of six animals contained developing worms. In addition, the peritoneal washings from infected animals showed evidence of a pronounced cellular infiltrate, consistent with the presence of worms.

### IVIS following footpad inoculation with L3

**Fig 2:**
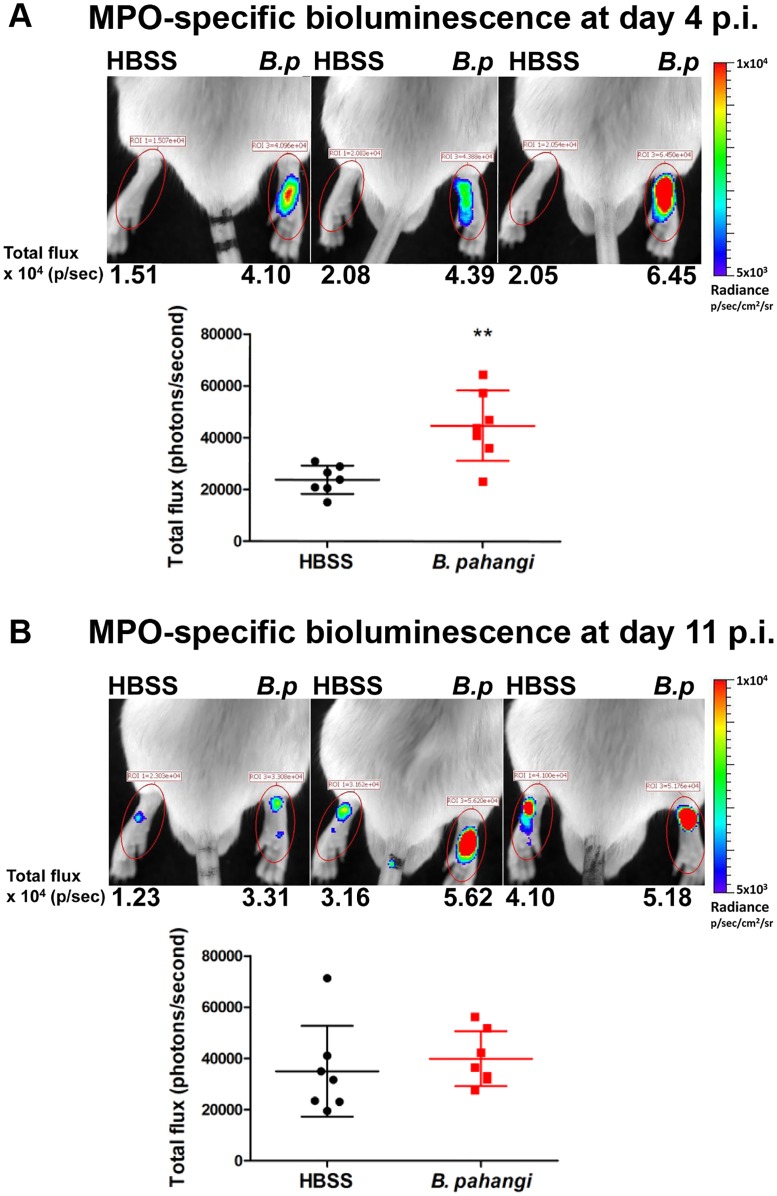
*In vivo* imaging of MPO-specific bioluminescence in mice infected with *B*. *pahangi* L3 larvae. BALB/c mice were injected with 50 L3 of *B*. *pahangi* into the RHS footpad (*B*.*p*) and with HBSS into the LHS footpad. On day 4 (**A**) and day 11 (**B**) post-infection mice were imaged using an IVIS spectrum 20 minutes after subcutaneous injection of 200 mg/kg luminol. Representative images of 3 mice per group are shown. The colour scale indicates bioluminescence radiance in photons/second/cm^2^/steradian. Graphs show the bioluminescence total flux (in photons/second) within the same footpad region of interest (ROI, red ovals in images). Each symbol shows the total flux for a single mouse, lines indicate the means (n = 7 mice) and error bars show SD (**p < 0.01 using a Mann-Whitney test).

As the results obtained with ip infections were not sufficiently discriminating, mice were then infected intradermally via the footpad, a route that leads to localisation of the worms in the lymphatic system [21]. In these experiments, mice received L3 into the RHS footpad with the LHS injected with HBSS as control. Imaging was carried out at various time points after injection of luminol sc into the nape of the neck. During the initial stages of infection (days 4–7), a specific luminescent signal was observed from the infected limb following injection of luminol. In the representative experiment shown in Fig 2 all seven mice showed stronger signal in the infected limb. Quantifying the signal showed a significant difference between infected and uninfected limbs (*p* = 0.0026, see Fig 2A). However, in all animals by day 11 p.i., the signal was reduced in the infected limb and there was no significant difference in myeloperoxidase-specific bioluminescence between uninfected and infected limbs at this time point (see Fig 2B). This lack of specificity at later time points was due to a reduction in signal from the infected limb but also a slight increase in signal and variability from the uninfected limb. This experiment was repeated with equivalent results.

**Fig 3:**
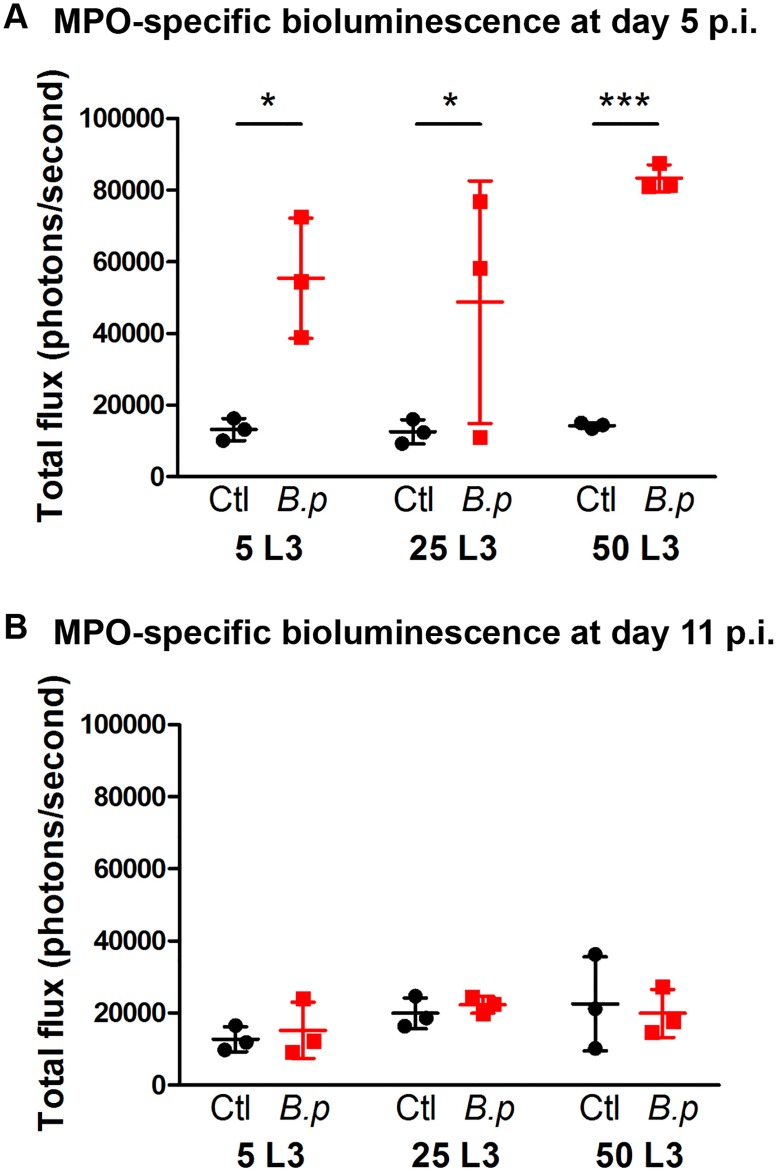
MPO-specific bioluminescence after infection with different doses of *B*. *pahangi*. BALB/c mice were injected with 5, 25 or 50 L3 of *B*. *pahangi* into the RHS footpad (*B*.*p*) and with HBSS into the LHS footpad (Ctl). On day 5 (**A**) and day 11 (**B**) post-infection mice were imaged using an IVIS spectrum 20 minutes after subcutaneous injection of 200 mg/kg luminol. Graphs show the bioluminescence total flux (in photons/second) within the footpad region of interest. Each symbol shows the total flux for a single mouse, lines indicate the means (n = 3 mice per L3 dose) and error bars show SD (*p < 0.05, ***p < 0.001 using two-way ANOVA with Bonferroni post-test).

In the next experiment, mice were injected with different numbers of L3 (5, 25 or 50 in the syringe) to investigate whether there was a correlation between the original inoculum and the intensity of the luminescent signal. As observed previously, at an early time point p.i. (day 5 in this experiment) the signal from each infected limb was much stronger than from the uninfected limb (*p* < 0.05 for 5 and 25 L3 and *p*<0.001 for 50 L3 compared to uninfected control limbs, see Fig 3A). However, the difference between infected and uninfected limbs was minimal by day 11 p.i. (see Fig 3B). Although the higher worm dose (50 L3) resulted in an elevated signal in all three mice, compared to the more variable signal observed between mice infected with lower numbers of worms, no significant correlation between the intensity of the signal at day 5 p.i. and the number of worms inoculated was observed.

### Histological observations

**Fig 4:**
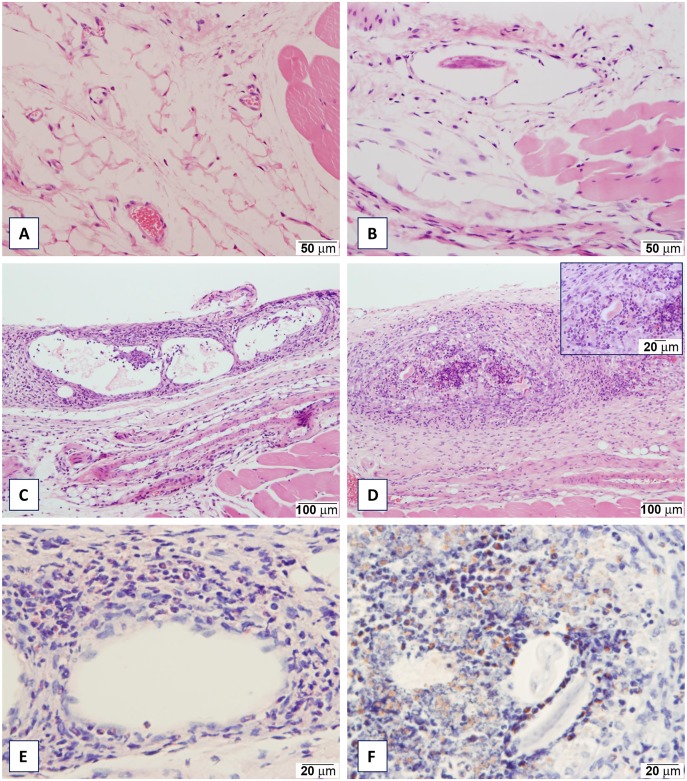
Histopathology of *B*. *pahangi* infected and uninfected limbs in a mouse model. BALB/c mice were injected with 50 L3 of *B*. *pahangi* into the RHS footpad and with HBSS into the LHS footpad and analysed on the days p.i. as indicated. **(A)** uninfected mouse, Day 21, HBSS-injected limb: there is no inflammation in the fascial plane **(B)** infected mouse, Day 7: a filarial nematode is present within the lumen of a dilated lymphatic vessel in the fascial plane. **(C)** infected mouse, Day 14 prominent lymphangiectasis and lymphangitis in the fascial plane. **(D)** infected mouse, Day 21: marked inflammatory infiltration obliterating the lumen of a lymphatic vessel. Inset: remnants of degenerate nematodes within the lumen surrounded by inflammatory cells. **(E)** infected mouse (same animal as section C), Day 14: the inflammatory infiltrate affecting the lymphatic vessel in the fascial plane is characterised by the presence of many eosinophils highlighted by the modified haematoxylin eosin method. **(F)** infected mouse, Day 21: nematodes surrounded by intense inflammatory infiltration comprising large numbers of eosinophils highlighted by the modified Congo Red protocol.

In order to ascertain whether the slight increase in luminescence at later time points in the uninfected limbs could be explained by migration of the L3 from the site of injection, histopathology was carried out on infected and uninfected limbs to confirm the presence of parasites and inflammation only in the infected limb. In the first experiment, a single time point was analysed at 21 days p.i. Here the difference was immediately visible between infected and uninfected limbs. The lymphatic vessels in the infected limbs were dilated, appeared to contain flocculent material and were often surrounded by inflammatory cells. In contrast, the uninfected limb showed no such changes (Fig 4A). Parasites were observed within the lumen of some lymphatic vessels, although whether these larvae were viable remains uncertain. In a subsequent experiment, histology was carried out over a time course of 7, 14 and 21 days p.i. with similar results (see Fig 4). At day 7, some animals showed minimal to mild interstitial inflammation in the subcutis or fascial planes. Nematode parasites were visible within the lumen of lymphatic vessels in the subcutis or fascial planes in some sections but there was no indication that parasites were present in uninfected limbs (Fig 4B). At 14 and 21 days p.i. there was an increased prominence of inflammatory changes in the fascial planes, and to a variable extent in the subcutis and deep dermis. In addition, in some animals a distinct pattern of perilymphangitis and lymphangitis was observed (Fig 4C and 4D). In some instances inflammatory cells were noted to cause complete disruption of the lymphatic vessel structure with obliteration of the lumen caused by accumulation of inflammatory cells and necrotic debris associated with remnants of degenerate parasites (Fig 4D). Inflammatory infiltrates in the subcutis and fascial planes, as well as those associated with lymphatic vessels, comprised variable proportions of eosinophils, neutrophils, lymphocytes and macrophages. Staining with the modified haematoxylin and eosin and Congo Red methods confirmed that eosinophils were the predominant cell type within the inflammatory infiltrate affecting the lymphatic vessels and surrounding remnants of parasites (Fig 4E and 4F).

### Cytokine analysis

**Fig 5:**
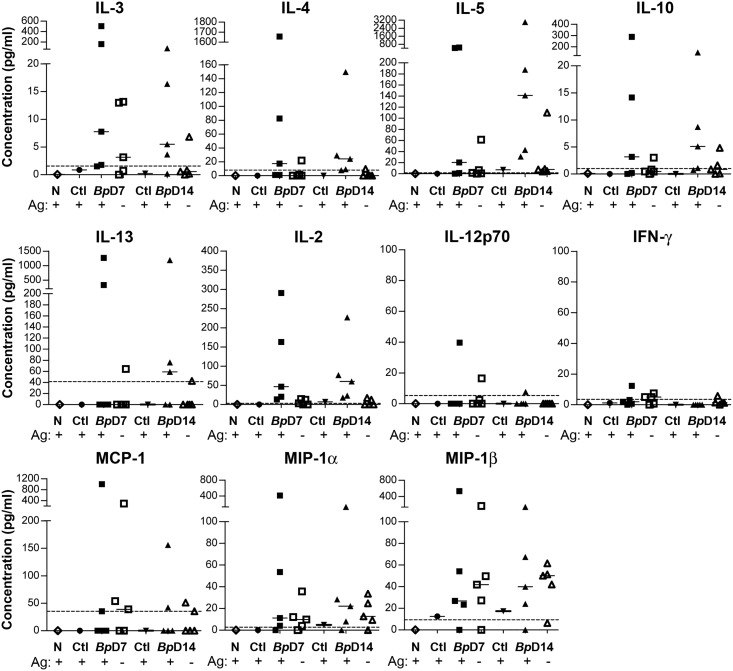
Cytokine production by lymph node cells after *in vitro* restimulation. BALB/c mice were injected with 50 L3 of *B*. *pahangi* into the RHS footpad and with HBSS into the LHS footpad. Popliteal LN cells were collected from infected limbs of individual mice at d7 (*Bp*D7) and d14 (*Bp*D14) post-infection and pooled from uninfected limbs (Ctl, ● d7, ▼d14 p.i.) or naive mice (N). Cells were re-stimulated with media (-) or 10 μg/ml *Brugia* antigen (+) as indicated for 48 h at 37°C, and the cytokine and chemokine production determined by Luminex assay. Values for individual mice are shown in samples from infected limbs and lines represent median values. The dotted line in each graph shows the limit of detection.

To further explore the immunological events associated with infection, the cytokine profile in antigen-stimulated popLN cells from both infected (RHS) and uninfected (LHS) limbs, as well as from naive mice, was assessed using bead array technology. As can be seen from Fig 5, the results were variable, with individual infected animals producing elevated levels of all cytokines at each time point assayed. However, some general points can be made: Th2-associated cytokine levels were higher overall, as might be expected given the nature of the infection; IL-3, IL-4, IL-5, IL-10 and IL-13, were all elevated in popLN cells from infected limbs, particularly so when re-stimulated with *Brugia* antigen. Individual mice that had high levels of one cytokine generally had higher levels of all other cytokines, suggesting these animals were particularly responsive. With the exception of IL-5, there was little difference in the median levels of Th2 cytokines between days 7 and 14 (see Fig 5); IL-5 levels increased between day 7 and day 14 in infected mice. While IL-2 levels were increased in responsive mice, most Th1-associated cytokines were either not detected, or were at very low levels (IL-12p70 and IFN-γ). Chemokines such as MCP-1 (CCL2), MIP-1α (CCL3) and MIP-1β (CCL4) tended to be higher in infected compared to uninfected limbs, and in addition MIP-1β was higher in popLN cells from the uninfected limb compared to naive mice.

## Discussion

Helminth parasites remain a major burden on human health in tropical regions of the world [22]. There are no vaccines available and control still relies upon the use of drugs, many of which were developed decades ago. In addition, the treatment of many helminth infections relies upon a single compound, giving rise to concerns about the possible development of anthelmintic resistance [23]. Several programmes are underway in an attempt to identify novel compounds suitable for human use, but the methodologies available for screening and testing large numbers of compounds, particularly *in vivo*, remain relatively limited. The aim of the work undertaken in this study was to determine if the significant progress made in imaging methods in live animals could be applied to assess the viability of filarial worms in a mouse model of infection. Specifically, we sought to determine whether the immune response elicited by living filarial worms could be visualised *in vivo* using reagents that emit bioluminescence when exposed to molecules released by immune cells. The best results were obtained by intradermal infection with L3, following which the parasites localise to the draining lymphatic vessels. However the luminescent signal was obvious only during the initial stages of infection as, by day 11–12 p.i., when the worms have developed to the L4 stage, there was no discrimination between infected and uninfected limbs.

The lack of specificity at later time points of infection could arise either because of the migration of developing larvae away from the site of infection or, alternatively, it could reflect the suppression of immune responses, a well-documented characteristic of lymphatic filarial nematodes [24,25,26]. Studies in the only fully permissive rodent host of *Brugia* species, the Mongolian jird, demonstrated that L3 rapidly migrated from the site of injection in the hind limb to the lymphatic system, with the majority of developing adults establishing in the lymphatics of the spermatic cord [27,28]. In the present study, we observed sections through filarial worms in the local lymphatic vessels of the infected limb at the latest time point examined (21 days p.i.) but never in uninfected limbs. To investigate whether a significant number of larvae had migrated from the infected limb, dissection of the whole animal would be necessary. In addition, the migratory capacity of the L3 may differ between a fully susceptible host (the jird) and a partially susceptible host (the BALB/c mouse). Varying the numbers of L3 injected did not result in a longer lasting inflammatory response as detectable by imaging. This observation accords with previous experiments that demonstrated that cytokine and proliferative responses were similar in mice given 5, 25 or 50 L3 of *B*. *pahangi* by the subcutaneous route [29].

The BALB/c mouse is semi-permissive for *B*. *pahangi* in as much as infection with L3 gives rise to only a few adult worms and these do not produce microfilariae. However L3 and developing L4 can be recovered in reasonable numbers at early time points of infection [30], as can transplanted adult worms [18]. This model was selected for imaging over the more permissive jird for several reasons: 1) the availability of established protocols for imaging, efficacy and pharmacokinetic studies in mice, 2) the smaller size of mice compared to jirds increases the imaging capacity and reduces the compound requirement for dosing, 3) use of inbred mice compared to outbred jirds reduces variability, thus allowing smaller group sizes, and 4) the availability of antibodies to mouse proteins and genetically engineered mouse strains expands the downstream applications of the model.

Infection via the footpad is known to elicit an early burst of IL-4 mRNA and significant levels of Th2 cytokines by day 7 of infection [20]. However, the ability of filarial worms to modulate host immune responses is well documented; the absence of a detectable inflammatory response using IVIS reagents at day 10–11 could reflect a progressive down-regulation of a pro-inflammatory response driven by the parasite. Cells from the draining lymph node secreted a range of cytokines, with higher levels of Th2 cytokines classically associated with filarial infection [24]. In addition, specific chemokines were detectable including MCP-1 (CCL2), MIP-1α (CCL3) and MIP-1β (CCL4). Chemokines may also reflect *in vivo* exposure to the *Wolbachia* endosymbiont of filarial worms [31]. However, chemokine levels were similar upon *in vitro* re-stimulation in the presence or absence of filarial antigen. Chemokines are critical signalling molecules that regulate leukocyte recruitment and trafficking in the context of inflammatory responses. While some cytokines/chemokines measured in this study peaked at day 7, histological evaluation of infected limbs demonstrated a time-dependent increase in the inflammatory response from day 7 to day 21 p.i. At 14 and 21 days p.i. inflammation was characterised by patterns consistent with lymphangitis and perilymphangitis, with infiltrates consisting of eosinophils, macrophages and lymphocytes, and lower numbers of neutrophils. In some instances the inflammatory infiltrates causing obliteration of the lymphatic vessels in the subcutis and fascial planes were clearly associated with remnants of degenerate nematode parasites. Consistent with the elevated levels of IL-5 secreted from popLN cells, eosinophils were particularly abundant in inflammatory foci; in addition, both MIP-1α and MIP-1β, which remained relatively stable over 2 weeks of infection, are known chemotactic factors for mouse eosinophils [32]. Previous studies have implicated eosinophils in killing both microfilariae [33] and later larval stages of filarial parasites [34]. In an elegant study in cattle naturally infected with the filarial worm *Onchocerca ochengi*, it was proposed that neutrophils, elicited by the *Wolbachia* endosymbiont, ‘protect’ the worm from lethal attack by eosinophils [35]. However, in our semi-permissive mouse model the most likely scenario is that infection with L3 induces a burst of cytokine/chemokine gene expression [20], which elicits trafficking of immune cells to the site of infection. While attrition of some of the nematodes follows, those left alive may then down-regulate immune responses. Such a scenario would correlate with the reduction in signal from IVIS imaging at later time points, and the relatively stable levels of some cytokines, despite the presence of a cellular infiltrate. As the L3 of filarial worms have known migratory capacity it was important to rule out whether the slight increase in background signal in mice infected with L3 might be explained by migration of the larvae into the uninfected limb. However, we could detect no evidence of L3 either by histology or by analysis of cytokine levels from the uninfected limb.

Imaging of the immune response limits this *in vivo* screening model to assessment of potential chemotherapeutic agents and highlights the desirability of developing imaging agents that could detect the worms themselves. If such reagents were available they could also be applied to vaccine studies or to efficacy studies involving immunomodulatory drugs. Filarial worms secrete a myriad of molecules under *in vitro* culture conditions, including enzyme activities such as triose phosphate isomerase, leucyl aminopeptidase and glutathione peroxidase [36,37]. More specific reagents are continually being developed to detect various disease states; for example, several reagents are already available that detect enzymatic activities of specific proteases, which are useful markers of pathologies such as arthritis, airway inflammation and tumor progression [38,39,40]. It may be possible in the future to utilise more specific reagents to detect secretory products of adult worms *in vivo*.

## Supporting Information

S1 Fig *In vivo imaging* of MPO-specific bioluminescence in mice injected ip with *B*. *pahangi* L3 larvae. BALB/c mice were injected ip with 50 L3 of *B*. *pahangi* (*B*.*p*) or with HBSS (Ctl). On day 7 (**A** and **B**), day 12 (**C**) and day 18 (**D**) post-infection mice were imaged using an IVIS spectrum without (**A**) or 20 minutes after (**B, C, D**) subcutaneous injection of 200 mg/kg luminol. Two to three representative mice from each group are shown. The colour scale indicates bioluminescence radiance in photons/second/cm^2^/steradian. (**E**) Graph shows the bioluminescence total flux (in photons/second) over the abdominal region of interest. Each symbol shows the total flux for a single mouse, lines indicate the means (n = 3–6 mice) and error bars show SD (*p < 0.05 using a Mann-Whitney test to compare background and MPO-specific bioluminescence at d7).
(TIF)
