# Supplementary figures and images for: Attempts to Image the Early Inflammatory Response during Infection with the Lymphatic Filarial Nematode Brugia pahangi in a Mouse Model
Source: PLoS One. 2016 Dec 16;11(12):e0168602. doi: 10.1371/journal.pone.0168602 (PMC5161388; doi:10.1371/journal.pone.0168602)

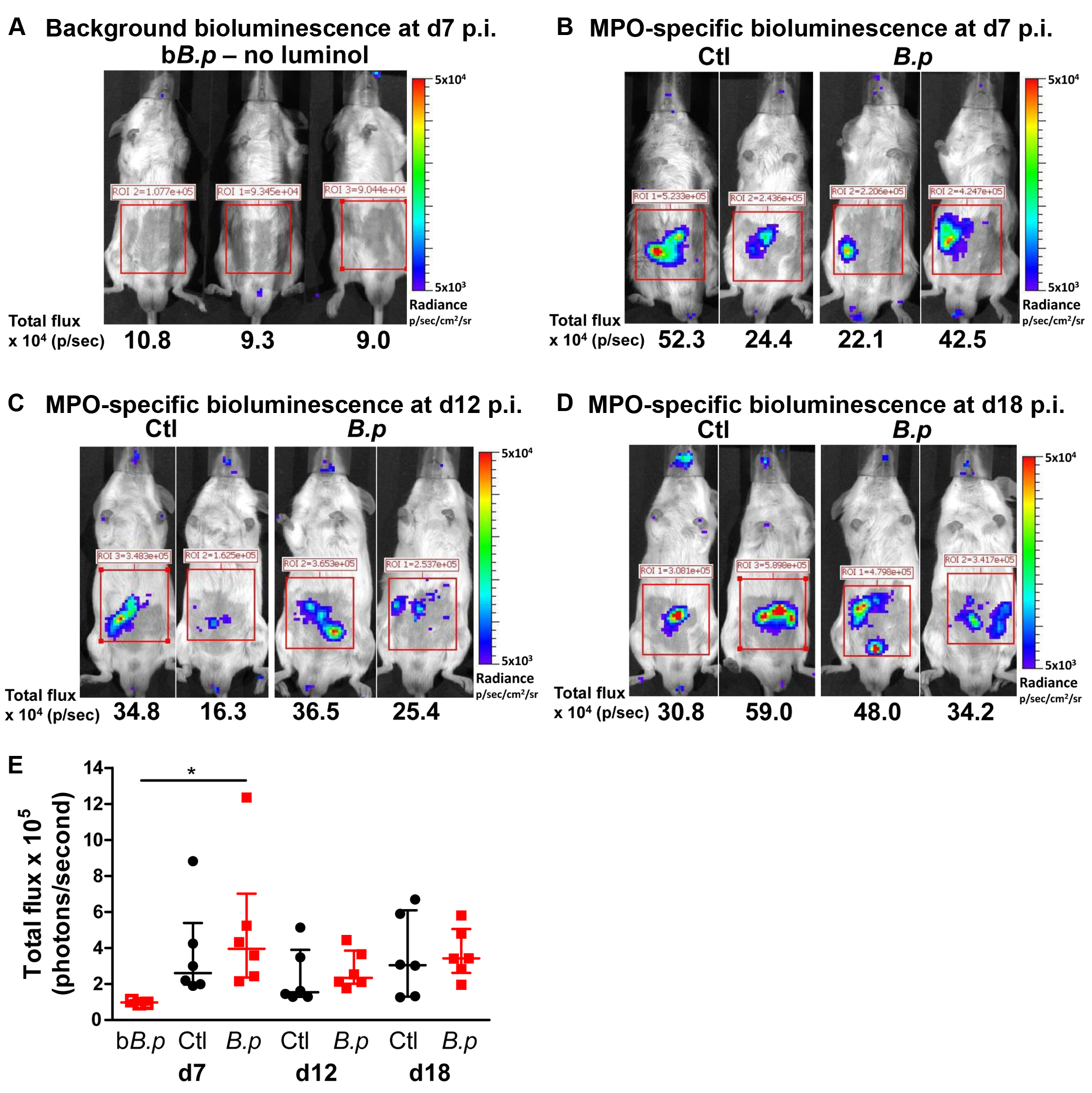

Supplement: S1 Fig — BALB/c mice were injected ip with 50 L3 of B. pahangi (B.p) or with HBSS (Ctl). On day 7 (A and B), day 12 (C) and day 18 (D) post-infection mice were imaged using an IVIS spectrum without (A) or 20 minutes after (B, C, D) subcutaneous injection of 200 mg/kg luminol. Two to three representative mice from each group are shown. The colour scale indicates bioluminescence radiance in photons/second/cm2/steradian. (E) Graph shows the bioluminescence total flux (in photons/second) over the abdominal region of interest. Each symbol shows the total flux for a single mouse, lines indicate the means (n = 3–6 mice) and error bars show SD (*p < 0.05 using a Mann-Whitney test to compare background and MPO-specific bioluminescence at d7). (TIF) [file pone.0168602.s001.tif]
